# Effects of Dietary Daidzein Supplementation on Reproductive Performance, Serum Hormones, and Reproductive-Related Genes in Rats

**DOI:** 10.3390/nu10060766

**Published:** 2018-06-14

**Authors:** Qiqi Zhang, Daiwen Chen, Bing Yu, Xiangbing Mao, Zhiqing Huang, Jie Yu, Junqiu Luo, Ping Zheng, Yuheng Luo, Jun He

**Affiliations:** 1 Institute of Animal Nutrition, Sichuan Agricultural University, Chengdu 611130, Sichuan, China; qqzhang6699@163.com (Q.Z.); dwchen@sicau.edu.cn (D.C.); ybingtian@163.com (B.Y.); xiangbingm@hotmail.com (X.M.); zqhuang@sicau.edu.cn (Z.H.); yujie@sicau.edu.cn (J.Y.); ljqlyh2009@yahoo.com.cn (J.L.); zpind05@163.com (P.Z.); luoluo212@126.com (Y.L.); 2 Key Laboratory for Animal Disease-Resistance Nutrition of China Ministry of Education, Chengdu 611130, Sichuan, China

**Keywords:** chemical parameters, reproductive-related genes, rats

## Abstract

The aim of this study was to investigate the effect of dietary daidzein supplementation on reproductive performance in rats. A total of twenty-four female Sprague–Dawley (SD) rats were randomly allocated to two groups and fed either with a basal diet (CON) or basal diet containing 50 mg/kg daidzein (DAI) from gestation until delivery stage. The results show that daidzein supplementation significantly increased the total litter weight and the total viable newborn weight (*p* < 0.05). Interestingly, daidzein supplementation acutely elevated the concentrations of serum estrogen, progesterone and insulin-like growth factor-1 (*p* < 0.01) after the maternal rats’ delivery. The concentrations of serum immunoglobulin A (IgA) and immunoglobulin G (IgG) were also significantly higher in the DAI maternal rats than in the CON maternal rats (*p* < 0.05). Moreover, daidzein significantly increased the total antioxidant capacity (T-AOC) in maternal rats’ sera and in newborns (*p* < 0.05) and elevated the concentration of superoxide dismutase (SOD) in both the maternal rats’ sera and their ovaries (*p* < 0.05). Importantly, daidzein supplementation significantly elevated the expression levels of estrogen receptor β (ERβ) and NR5A2 genes in maternal rats’ ovaries (*p* < 0.05) and downregulated the expression level of prolactin receptor (PRLR) in newborns (*p* < 0.05). These results suggest that dietary daidzein supplementation improves reproductive performance and fetal development in rats, which is associated with changes in serum hormones, tissue antioxidant capacity, and expression levels of reproductive-related genes, both in maternal rats and their offspring.

## 1. Introduction

Daidzein is a kind of natural isoflavonic phytoestrogen with estrogenic activity, and its activity is about 10^–3^–10^–5^ times that of 17 β-estradiol [1]. Daidzein is isolated mainly from natural products, such as beans, pasture grasses and cereals. Due to the characteristics of the estrogenic activity of daidzein, it can directly bind to the estrogen receptors to varying degrees and regulate the hypothalamic–pituitary–gonadal axis of the neuroendocrine system of animals [2].

Previous studies have indicated that daidzein has beneficial effects on animal growth and health [3]. In recent years, as a dietary component, daidzein has received much attention due to its potential effects on animal fertility, and the supplementation of daidzein to animals during pregnancy has been extensively studied. Research has demonstrated that sows supplemented with daidzein at a dose of 0.005 mg/kg for 30 days before parturition until seven days after birth resulted in significant increases in litter weight at birth and the 20-day litter weight of piglets, as well as milk production within 10 days after delivery, and levels of growth hormone, insulin-like growth factor-1 (IGF-1) and thyroid stimulating hormone (TSH) in colostrum [4]. Furthermore, multiparous sows have been supplemented with dietary daidzein of 1 mg/kg body weight per day during late gestation, which could affect meat quality and skeletal muscle cellularity of the multiparous sow’s offspring [5]. Concerning poultry subjects, there have also been studies that have shown that daidzein could be a very effective additive to improve laying performance, eggshell quality and egg yolk total superoxide dismutasein in laying hens during the late laying period [6]. Another study showed that daidzein supplementation could increase egg weight and fertility, and affect serum progesterone (P4), thyroxine (T4) and growth hormone (GH) concentrations in female geese [7]. Studies have shown that the addition of daidzein (100 ppm) to the diet of pregnant rats enhances the wet weight and mammary gland development. Moreover the milk yield and serum growth hormone (GH) and prolaction (PRL) levels as well as both the number and affinity of the mammary cytosol estradiol receptors increased markedly [8]. Previous research showed that the dams’ body weights and litter weights at birth were significantly decreased when the Sprague–Dawley (SD) rats were fed a diet containing 0.50 g/kg of daidzein during pregnancy and lactation [9]. Supplementation of virgin female rats with 250 mg and 1000 mg daidzein/kg diet from two weeks prior to breeding until day 50 postpartum had no significant effect on fertility, numbers of male and female offspring, or anogenital distances. Additionally, both daidzein doses slightly, but not significantly, decreased ovarian and uterine weights, as well as mammary gland sizes [10]. Daidzein is also reported to have antioxidant, antimicrobial, and anti-inflammatory health benefits [11,12,13]. Although a lot of research has been performed, there is no clear-cut evidence to show the exact relationship between the dose and effects of daidzein, since the effects of daidzein on animals are closely associated with the animal species, sex, dosage and the physiological stage of animals. Moreover, the mechanisms behind the daidzein-regulated biological events still remain unclear, for now.

Given the above considerations, dietary daidzein supplementation is potentially beneficial for animal reproduction. The aim of this study is to investigate the effect of daidzein supplementation at a moderate dose of 50 mg/kg from gestation until the delivery stage on the reproductive performance of rats. Furthermore, the authors determine serum immunoglobulins, sera and tissue antioxidant indices in rats to assess the effect of daidzein on rats’ immunity and antioxidant statuses. Moreover, several critical genes involved in reproductive functions are determined to explore potential molecular mechanisms behind the daidzein regulated reproductive performance in rats.

## 2. Materials and Methods

### 2.1. Animal and Experimental Diets

All animal procedures were approved by the Animal Care and Use Committee of Sichuan Agricultural University. They were implemented based on the National Research Council’s Guide for the Care and Use of Laboratory Animals. The thirty-six 70–90-day-old virgin female Sprague–Dawley rats (250–270 g body weight) used in the current research were purchased from Chengdu Dashuo Experimental Animals Co., Ltd. (Chengdu, China). All rats were housed individually in clear stainless-steel cages at a temperature of 22–24 °C, humidity of 50–60%, and a 12-h/12-h light/dark cycle-controlled environment. Diets and water were provided ad libitum.

After 7 days of acclimatization with a basal diet, female rats were mated with male rats until pregnancy, which was confirmed by the method of vaginal smear. Twenty-four pregnant rats were allocated at random to two groups (control group and daidzein group, n = 12). Rat body weights were recorded at this point. Pregnant rats received either a normal control diet (supplementation with 0 mg daidzein/kg feed) or a daidzein diet (supplementation with 50 mg daidzein/kg feed). The daidzein was purchased from Sigma. (purity ≥ 98%, Beijing, Mainland, China). The authors mixed the daidzein into the basal diet powder and then pelletized it manually. The pellets were dried in the oven at 50 °C. Approximately 21 days later, the rats delivered and then were sacrificed for the collection of blood and organs for analysis.

### 2.2. Sample Collection

Following delivery, the maternal rats’ and newborns’ body weights were recorded. Subsequently, the maternal rats were anesthetized using anhydrous ether, and blood samples were drawn from the abdominal aorta into a serum separator tube. Maternal blood was centrifuged at 3500 rpm for 15 min at 4 °C to obtain sera, and then the sera was stored at −20 °C until analysis. Maternal uteri and ovaries were dissected, rinsed with phosphate buffered saline (PBS), dried with filter paper, and weighed with an analytical balance. Then, all samples were immediately frozen in liquid N_2_ and stored at −80 °C until analysis.

### 2.3. Measurements and Analytical Methods

#### 2.3.1. Reproductive Performance

The maternal initial pregnancy body weights were recorded once it was determined that the rats were pregnant. Moreover, the maternal postpartum body weight, maternal body weight gain, daily food intake from gestation until delivery stage, number of total/live newborns per litter, individual newborn weights, total/viable/inviable newborn weight per litter, individual newborn lengths, uterine weights and ovarian weights were recorded. Individual newborn length was measured with Vernier calipers. Additionally, the newborn survival rate and reproductive organ index were calculated as follows:

newborn survival rate (%) = live newborns (n)/total newborns (n) × 100%,(1)

reproductive organ index (mg/g) = reproductive organ weight (mg)/body weight (g).(2)

#### 2.3.2. Serum Reproductive Hormones

Maternal serum estrogen (E), progesterone (P) and insulin-like growth factor-1 (IGF-1) levels were determined by enzyme-linked immunosorbent assay (ELISA) kits. The kits used for testing were purchased from Beijing Donggeboye Biological Technology Co., Ltd. (Beijing, China), and the detailed operations were as per the kits’ instructions.

#### 2.3.3. Serum Immunoglobulins

The contents of IgA and IgG in the maternal rats’ sera were detected by ELISA kits (Beijing Donggeboye Biological Technology Co., Ltd., Beijing, China). All steps were performed according to the manufacturer’s instructions.

#### 2.3.4. Serum Metabolites

The concentration of glucose (GLU), total protein (TP), albumin (ALB), urea nitrogen (BUN), aspartate aminotransferase (AST), alanine aminotransferase (ALT), triglyceride (TG), total cholesterol (TC), high-density lipoprotein cholesterol (HDL-C) and low-density lipoprotein cholesterol (LDL-C) in maternal rats’ sera were determined using commercially available reagent kits purchased from Nanjing Jiancheng Bioengineering Institute (Nanjing, China). The glucose oxidase peroxidase method was used for detection of the concentration of GLU, the Bradford method was used for the test for the TP content, the colorimetric method was used to determine the concentration of ALB (with standard: bromocresol green method), the BUN content was determined by the Urease method, the Reitman’s method was used for the determination of AST and ALT activities, and TG and TC concentrations were determined by the single reagent GPO-PAP method. The determination of HDL-C and LDL-C concentrations was done with a double reagent direct method. All operations were in accordance with the instructions of the kits.

#### 2.3.5. Antioxidant Indices of Maternal Rats’ Sera, Uteri, Ovaries, and Fetal Longissimus Dorsi Muscles

The total antioxidative capability (T-AOC), total superoxide dismutase (T-SOD), catalase (CAT), glutathione peroxidase (GSH-Px) and malondialdehyde (MDA) content in maternal rats’ sera, uteri, ovaries and fetal longissimus dorsi muscle samples were measured using kits from Nanjing Jiancheng Bioengineering Institute (Nanjing, China) in accordance with the manufacturers’ instructions on the kits. The activities of T-AOC and GSH-Px were detected by colorimetric assay. The T-SOD, CAT and MDA contents were determined by the hydroxylamine, visible light and thiobarbituric acid methods, respectively.

#### 2.3.6. RNA Extraction and Quantitative Real-Time PCR of Maternal Rats’ Uteri, Ovaries, and Fetal Longissimus Dorsi Muscles

Total RNA was extracted from maternal rats’ uteri, ovaries and fetal longissimus dorsi muscle samples using the TRIzol reagent (Takara, Dalian, China) in accordance with the manufacturer’s guidance. RNA integrity was detected using agarose gel electrophoresis, and concentration and purity were measured on a UV/VIS spectrophotometer (Beckman Coulter, DU720). RNA purity was determined by measuring the A260/A280 ratios. Complementary DNA (cDNA) was synthesized from 1 µg of RNA via reverse transcription using an RNeasy Mini Kit (Takara) as per the kit’s instructions. The cDNA was subjected to quantitative real-time PCR using DNA-specific primers. β-actin (Sangon Biotech, Shanghai, China) was utilized as the housekeeping reference gene, and the primers (Sangon Biotech) for the target genes are listed in Table 1. The genes of interest were amplified on a Thermal Cycler (CHRMO4-TM Thermal Cycler; Bio-Rad, Inc., Hercules, CA, USA) according to the manufacturer’s protocol. The PCR conditions were as follows: 95 °C for 30 s, followed by 40 cycles at 95 °C for 5 s, 60 °C for 34 s, under melt curve conditions at 95 °C for 15 s, 60 °C for 1 min, and then 95 °C for 15 s (temperature change velocity 0.5 °C s). Additionally, data were quantified using the ΔΔCt method, as described previously [14]. The expression of the following genes was analyzed: prolactin receptor (PRLR), estrogen receptor α (ERα), estrogen receptor β (ERβ), liver receptor homologue-1 (LRH-1/NR5A2), transforming growth factor-alpha (Tgf-α), and epidermal growth factor (EGFR).

### 2.4. Statistical Analysis

All data was presented as means ± standard deviations (SD). Statistical analyses were performed using SPSS 20.0 software (IBM, SPSS, Chicago, IL, USA). All results were analyzed by Student’s *t* tests, except the effects of daidzein supplementation on individual newborn weight, serum estrogen and progesterone levels, which were analyzed by using a General Linear Model (GLM) with the Univariate option with the litter size was used as a covariate. *p*-Values less than 0.05 were considered statistically significant.

## 3. Results

### 3.1. Reproductive Performance as Affected by Daidzein

Table 2 shows that compared with the control group, daidzein supplementation significantly increased the total litter weight (*p* < 0.05) and the total viable newborn weight (*p* < 0.05). The numbers of total newborns and live newborns were not significantly different between the two groups (*p* > 0.05). Additionally, the ANCOVA results showed that, after the control of the litter size was used as a covariate, daidzein supplementation had no effect on individual newborn weight (*p* = 0.769).

### 3.2. Effects of Daidzein on Sera Hormone Levels in Maternal Rats

Table 3 shows that supplementation with 50 mg/kg daidzein in the diet significantly increased the levels of serum estrogen, progesterone and insulin-like growth factor-1 in maternal rats (*p* < 0.01). The ANCOVA results show that after adding the control litter size as a covariate, daidzein supplementation had significant effects on estrogen levels and progesterone levels.

### 3.3. Effects of Daidzein on Serum Immunoglobulin Levels in Maternal Rats

Table 4 shows that, after adding 50 mg/kg daidzein into the diet, the serum immunoglobulin G levels of maternal rats were significantly higher than those of the control group (*p* < 0.01) and the levels of immunoglobulin A were also increased (*p* < 0.05).

### 3.4. Effects of Daidzein on Sera Metabolite Levels in Maternal Rats

Table 5 shows that, compared with the control group, supplementation with 50 mg/kg of daidzein in the diet increased the activities of T-AOC and SOD (*p* < 0.05) and tended to decrease the activity of MDA, while the activities of CAT and GSH-Px were not affected (*p* > 0.05). Additionally, changes in other sera metabolite indices were not significant (*p* > 0.05).

### 3.5. Effects of Daidzein on Antioxidant Indices in Maternal Rats’ Uteri, Ovaries, and Fetal Longissimus Dorsi Muscles

Table 6 shows that, compared with the control group, supplementation with daidzein increased the activities of SOD in maternal rats’ ovaries (*p* < 0.05) and T-AOC in fetal longissimus dorsi muscles (*p* < 0.05), and tended to increase the activity of SOD in maternal rats’ uteri.

### 3.6. Relative Expression Levels of Related Genes in Maternal Rats Uteri, Ovaries, and Fetal Longissimus Dorsi Muscles

**Figure 1:**
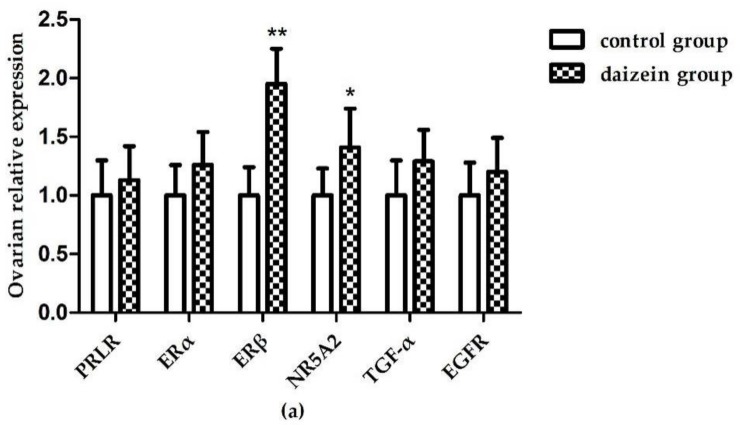

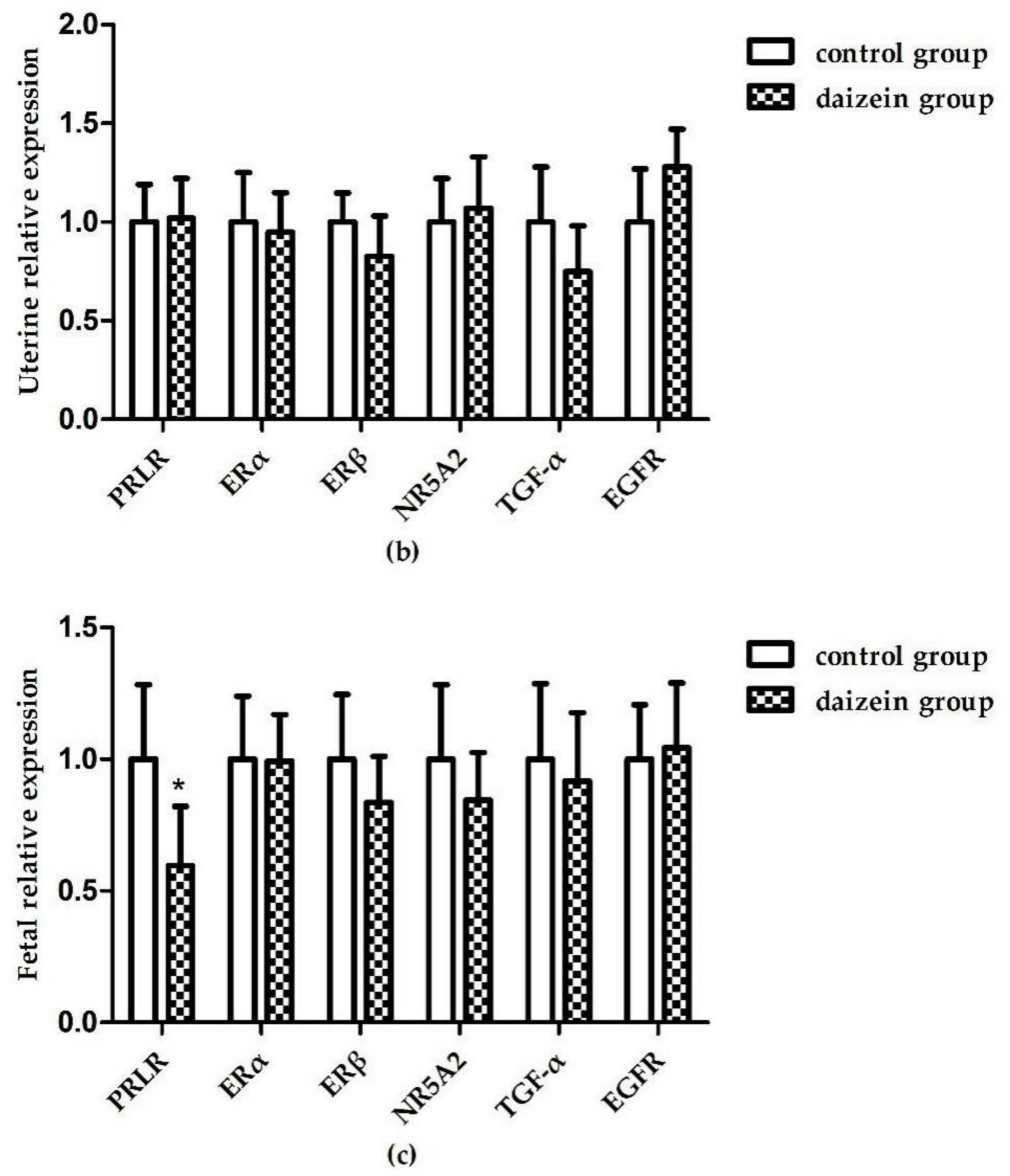
Relative gene expression of PRLR, ERα, ERβ, NR5A2, TGF-α, and EGFR in maternal rats’ ovaries (**a**) and uteri (**b**) and fetal longissimus dorsi muscles (**c**) (n = 12). Gene expression data were calculated relative to the control group’s data and normalized to the house-keeping gene, β-actin. The error bars represent 95% confidence intervals. (** *p*-value < 0.01, * *p*-value < 0.05 versus control group).

Figure 1a shows that, compared with the control group, the relative expressions of ERβ and NR5A2 mRNA in maternal rats’ ovaries increased significantly (*p* < 0.05) after daidzein supplementation. The relative expression of ERβ mRNA in the ovaries was significantly higher than that in the control group (*p* < 0.01). However, there was no significant difference in the mRNA expression of the other genes between two groups (*p* > 0.05). Although the expression of EGFR mRNA increased in the uteri, the difference was still not significant (*p* = 0.065, Figure 1b). In the fetal longissimus dorsi muscles (Figure 1c) the relative expression of PRLR mRNA decreased significantly (*p* < 0.05) compared with the control group.

## 4. Discussion

The current authors found that daidzein supplementation significantly increases the total litter weight and the viable newborn weight per litter (*p* < 0.05). This is probably due to the increased number of total live newborns (11.83 versus 16.17). These results are consistent with a previous report, in which daidzein treatment significantly increased the birth weights of male piglets [15]. Some other studies have also presented favorable results showing that daidzein promotes animal growth performance [3,4,16]. Additionally, the number of total newborns and live newborns showed an increasing trend in the daidzein group. Since the maternal rats were fed the experimental diet after mating, the effects of the experimental diet on ovulation in maternal rats were excluded; hence daidzein supplementation probably reduces the reabsorption of embryos during embryonic development to increase the number of newborns. These results suggest that supplementation with 50 mg/kg daidzein in the diet might be beneficial for fetal growth development, thus leading to increased fetal birth litter weights and litter sizes. This is possibly because daidzein binds to estrogen receptors causing an estrogenic effect to improve rats’ reproductive performances. Additionally, daidzein can also act as an antagonist that competitively binds to estrogen receptors, resulting in a weaker estrogen effect when endogenous estrogen levels are higher [17]. More research is needed to investigate the effect of diadzein on rats’ reproductive performances.

It is explicit that IGF-1 plays a major role in controlling body growth [18,19]. Previous studies have shown that exogenous estrogens can stimulate tissue and cell growth [20,21]. In the current study, daidzein supplementation increased the serum IGF-1 levels of maternal rats which is consistent with a previous study in which daidzein elevated serum IGF-1 levels in male piglets [3]. Moreover, daidzein may enhance serum GH and IGF-1 levels in bull calves [22]. However, the exact mechanism causing these results is complex and remains unclear, but it is partially correlated with the estrogen modulating growth-axis functions [15] since daidzein shows the estrogenic activity. Furthermore, supplementation of sows with daidzein during late gestation was found to stimulate fetal growth and promote IGF-1 receptor gene expression in newborn piglets [15]. Combined with the current study’s results, this indicates that daidzein supplementation could increase serum IGF-1 levels, thereby influencing the gestational growth of maternal rats and might further indirectly facilitate fetal growth through maternal nutrition transmission.

Researchers have reported that the estrogenic activity of daidzein affects the hypothalamic–pituitary–gonad axis of the neuroendocrine system by competitively combining with estrogen receptors to alter endogenous hormones levels and further regulating reproductive function [2]. The concentrations of progesterone and estrogen during pregnancy are high in mammals. Progesterone is critical for the establishment and maintenance of pregnancy, as its functions support ovulation and uterine as well as mammary gland development. The ovarian corpus luteum is the major source of progesterone during pregnancy [23]. Estrogen not only plays an essential role in the maintenance of pregnancy [24], but also participates in the implantation process and stimulates the development of the mammary gland [25,26]. Generally, estrogen and progesterone perform in synergy. In the present study, daidzein supplementation significantly improved the serum estrogen and progesterone levels, which suggested that the daidzein improveed reproductive performance in rats is associated with higher levels of hormones. The authors also believe that these increased hormones levels might result in part from the exogenous daidzein, since it showed some estrogen activity after absorption into the body, as demonstrated in the ANCOVA results in Table 3. Research has shown that daidzein increases serum estradiol and progesterone levels in middle-aged female rats [27]. In another study, daidzein increased serum progesterone concentrations, but had no effect on oestradiol concentrations, in geese [7]. There have also been studies indicating that daidzein decreases serum estradiol levels in female rats [28]. The effects of daidzein on the blood sex hormone levels in female rats remains an open question. Domestic and foreign researchers have come to different conclusions, which have been mainly related to the exposure time and dosage of the tested animals.

The results of this study show that daidzein significantly enhances the levels of serum immunoglobulin A and immunoglobulin G. A previous study showed that daidzein, at doses of 20 and 40 mg/kg, enhanced several immunologic functions of mice, such as the phagocytic response of peritoneal macrophages, the thymus weight, the quantity of spleen immunoglobulin M-producing cells against sheep red blood cells, and the lymphocyte proportion in peripheral blood [29]. It was clearly manifested that daidzein can reinforce nonspecific immunity, humoral immunity, and cell-mediated immunity. Another study demonstrated that daidzein can increase the levels of IgG, interferon alpha (IFN-α) and interleukin-2 (IL-2) in late lactation cows [30]. Therefore, the current study’s results are consistent with previous studies.

There has been plentiful research showing that daidzein could enhance antioxidant activity of organisms; the current study also proved this. The results show that supplemental daidzein increased the sera activities of T-AOC and SOD and tended to decrease the activity of MDA. Furthermore, it increased the activities of SOD in maternal rats’ ovaries and T-AOC in the fetal longissimus dorsi muscles and tended to increase the activity of SOD in maternal rats’ uteri. These observations indicate that daidzein could improve the antioxidant statuses of maternal rats and their offspring, which is consistent with previous studies that have suggested that daidzein can increase the activities of SOD, CAT, and GPx in rats’ mammary glands and liver tissues [8]. Furthermore, studies have shown that daidzein possesses free radical-scavenging properties, whereby its chemical groups react directly with free radicals to terminate the chain reactions of free radicals [31,32]. Therefore, daidzein can directly or indirectly strengthen the antioxidant activity of an animal, and the enhanced antioxidative potential is probably attributable to the nature of daidzein [8]. The effects of daidzein on the antioxidant status in the animal body might be related to the dosage, animal species, physiological stage of an animal and exposure time. Any of these factors might affect the measured parameters in the tissues. Hence, more tissue samples need to be collected in a new study to validate this.

Several critical genes involved in animal reproductive functions were determined. The prolactin receptor (PRLR), is a member of the cytokine receptor superfamily that initiates signal transduction pathways and is widely expressed in mammalian mammary glands, corpera lutea, ovaries, testicles, prostates and more. Research has indicated that PRLR is associated with female fertility, and doesnot only promote breast development and stimulate lactation [33], but also may be connected with fetal development [34,35]. Daidzein downregulated PRLR mRNA levels in the fetal *longissimus dorsi* muscles, which is consistent with a previous study that suggested that daidzein could downregulate PRLR mRNA levels in the geese pituitary during laying and broodiness [7]. The estrogen receptor (ER), a nuclear receptor belonging to the superfamily of steroid hormone receptors, can mediate estrogens so that they realize their capabilities. Until now, two subunits, ERα and ERβ, have been discovered and confirmed to exist; they are encoded by different genes on different chromosomes. In this study, daidzein supplementation upregulated the relative expression of ERβ mRNA in maternal rats’ ovaries, which is consistent with a previous study that reported that daidzein increases the expressions of ERβ mRNA and proteins on the porcine ovary [36]. There have also been studies revealing that daidzein decreases the expression of both ERα and ERβ mRNA in mouse ovaries [37]. The differences between these results might be related to species, administration methods, and exposure periods. The nuclear receptor subfamily 5, group A, member 2 (NR5A2), also known as the liver receptor homologue 1 (LRH-1) and the fetoprotein transcription factor (FTF), plays an important role in cholesterol metabolism, steroidogenesis, embryogenesis, and progesterone synthesis [38,39]. It is mainly expressed in the liver, ovaries, intestines and pancreas, and in particular, are expressed highly in ovarian granulosa cells and corpora lutea [39]. NR5A2 regulates progesterone synthesis in the ovaries and their actions in the uterus [39]. A recent study showed that mice lacking NR5A2 in granulosa cells are infertile and have substantially reduced progesterone levels [40]. Furthermore, previous research has proven that NR5A2 is necessary for maintenance of the corpus luteum, for promotion of decidualization and for placental formation; therefore, it is indispensable in establishing and sustaining pregnancy [41]. Recently, NR5A2 was shown to participate in early embryogenesis, and is expressed strongly in the embryonic endoderm, the developing liver, intestine, and the pancreas [42]. The relative expression of NR5A2 mRNA in the maternal rats’ ovaries increased significantly after daidzein supplementation in the current study. These results imply that 50 mg/kg daidzein supplementation from gestation until the delivery stage might facilitate embryogenesis and progesterone synthesis by upregulating the relative expression of NR5A2 mRNA in the maternal rats’ ovaries, thereby resulting in improvements in maternal rats’ serum progesterone levels and fetal development.

## 5. Conclusions

To conclude, the results of this study indicated that daidzein supplementation at a dose of 50 mg/kg from gestation until the delivery stage is beneficial for reproductive performance and fetal development in rats. Such beneficial effects might be associated with an improvement in sera hormone levels, immunoglobulin concentrations, antioxidant capacity, and the expression of reproductive-related genes. This study offers a molecular basis understanding the potential mechanisms behind the improved reproductive performance in rats by daidzein supplementation.

## Figures and Tables

**Table 1:** Primers for real-time quantitative PCR.

Genes	Primer Sequences	Size/bp	Accession Number	Annealing Temperature/°C
PRLR	F: CTACTTCTGACTGTGAGGACTTGCTG R: GGCTTAACACCTTGACCTGGATACTC	112	NM_001034111	59
ERα	F: TCTGGAGTGTGCCTGGTTGGAG R: GCGGAATCGACTTGACGTAGCC	175	NM_012689	61
ERβ	F: TCACGTCAGGCACATCAGTAACAAG R: CATCTCCAGCAGCAGGTCATACAC	94	NM_012754	60
NR5A2	F: GTCTCAGGTGATCCAAGCGATGC R: AGTCTGTCGGAGGCAAGGCTAC	110	NM_021742	61
Tgf-α	F: CCCTCCTGAAAGGAAGGACTG R: AGACACCTTTCCTTGGTTGGG	111	NM_012671	58
EGFR	F: AATCCTTGATGAAGCCTACGTGATGG R: TGGACAGTGGAGGTCAGACAGATG	87	NM_031507	60
β-actin	F: TGTCACCAACTGGGACGATA R: GGGGTGTTGAAGGTCTCAAA	165	NM_031144.3	60

F: forward; R: reverse; prolactin receptor (PRLR); estrogen receptor α (ERα); estrogen receptor β (ERβ); transforming growth factor-alpha (Tgf-α); epidermal growth factor (EGFR).

**Table 2:** Reproductive performance of maternal rats fed a basal diet supplemented or not supplemented with 50 mg/kg daidzein.

Parameters	Control Group	Daidzein Group	*p*-Value
Maternal initial pregnancy body weight (g)	274.50 ± 10.90	275.53 ± 9.75	0.866
Maternal postpartum body weight (g)	343.03 ± 36.08	340.42 ± 20.53	0.881
Maternal body weight gain (g)	68.53 ± 26.59	64.89 ± 12.94	0.769
Total newborns (n)	12.17 ± 3.87	16.50 ± 3.73	0.076
Live newborns (n)	11.83 ± 3.54	16.17 ± 3.82	0.069
Individual newborn weight (g)	6.37 ± 0.67	6.20 ± 0.83	0.769
Individual newborn length (mm)	42.71 ± 4.32	43.71 ± 4.36	0.143
Total litter weight (g)	75.35 ± 17.49	101.34 ± 14.04	0.018
Total viable newborn weight (g)	75.33 ± 17.46	101.32 ± 14.05	0.018
Total inviable newborn weight (n)	0.33 ± 0.52	0.33 ± 0.52	1.000
Uterine weight (g)	3.65 ± 0.84	4.28 ± 0.82	0.222
Ovarian weight (g)	0.16 ± 0.02	0.16 ± 0.03	0.891
Uterus index	10.64 ± 2.26	12.56 ± 2.30	0.175
Ovary index	0.46 ± 0.04	0.46 ± 0.07	0.963
Newborn survival rate (%)	97.85 ± 3.34	97.69 ± 3.83	0.942
Food intake (g/day)	18.98 ± 1.29	20.18 ± 1.02	0.104

Values are means ± SDs of 12 animals per group.

**Table 3:** Sera hormone levels of maternal rats fed a basal diet supplemented or not supplemented with 50 mg/kg daidzein.

Parameters	Control Group	Daidzein Group	*p*-Value
Estrogen (pg/mL)	106.23 ± 14.60	136.79 ± 14.59	<0.01
Progesterone (ng/mL)	10.04 ± 1.47	11.37 ± 0.53	<0.01
Insulin-like growth factor-1 (ng/mL)	1093.23 ± 187.33	1444.64 ± 182.20	<0.01

**Table 4:** Serum immunoglobulins levels of maternal rats fed a basal diet supplemented or not supplemented with 50 mg/kg daidzein.

Parameters	Control Group	Daidzein Group	*p*-Value
IgA (μg/mL)	159.96 ± 15.47	184.38 ± 18.72	0.034
IgG (μg/mL)	370.03 ± 44.99	498.55 ± 50.76	<0.01

Immunoglobulin A (IgA); immunoglobulin G (IgG)

**Table 5:** Sera metabolite levels of maternal rats fed a basal diet supplemented or not supplemented with 50 mg/kg daidzein.

Parameters	Control Group	Daidzein Group	*p*-Value
Glucose (mmol/L)	7.65 ± 1.44	8.63 ± 2.10	0.369
Urea nitrogen BUN (mmol/L)	10.91 ± 2.28	11.69 ± 2.63	0.597
Total protein (g/L)	35.84 ± 6.81	32.1725 ± 8.56	0.431
Albumin protein (g/L)	21.87 ± 4.60	19.62 ± 3.36	0.357
ALT (U/L)	22.99 ± 9.49	24.65 ± 9.12	0.765
AST (U/L)	30.47 ± 3.47	31.16 ± 4.48	0.774
Triglycerides (mmol/L)	1.46 ± 0.67	1.64 ± 1.05	0.720
Total cholesterol (mmol/L)	3.31 ± 0.52	3.26 ± 0.36	0.864
HDL-C (mmol/L)	1.78 ± 0.37	1.71 ± 0.21	0.693
LDL-C (mmol/L)	0.74 ± 0.31	0.96 ± 0.50	0.396
T-AOC (U/mL)	6.32 ± 0.82	8.17 ± 1.68	0.036
SOD (U/mL)	161.08 ± 8.23	175.26 ± 6.11	<0.01
CAT (U/mL)	12.08 ± 5.02	15.10 ± 5.77	0.357
GSH-Px (U/mL)	1403.91 ± 107.04	1463.30 ± 87.47	0.317
MDA (nmol/mL)	10.96 ± 2.16	8.72 ± 1.67	0.073

Urea nitrogen (BUN), aspartate aminotransferase (AST), alanine aminotransferase (ALT), total cholesterol (TC), high-density lipoprotein cholesterol (HDL-C) and low-density lipoprotein cholesterol (LDL-C); The total antioxidative capability (T-AOC), total superoxide dismutase (T-SOD), catalase (CAT), glutathione peroxidase (GSH-Px); malondialdehyde (MDA)

**Table 6:** Antioxidant indices of maternal rats’ uteri, ovaries, and fetal longissimus dorsi muscles after consumption of a basal diet supplemented or not supplemented with 50 mg/kg daidzein.

Parameters	Control Group	Daidzein Group	*p*-Value
Uterus			
T-AOC (U/mg protein)	1.39 ± 0.46	1.79 ± 0.60	0.224
SOD (U/mg protein)	25.92 ± 4.93	33.12 ± 8.20	0.095
CAT (U/mg protein)	66.50 ± 12.40	71.62 ± 10.74	0.462
GSH-Px (U/mg protein)	1564.87 ± 191.16	1691.88 ± 238.83	0.333
MDA (nmol/mg protein)	1.39 ± 0.46	1.79 ± 0.60	0.224
Ovaries			
T-AOC (U/mg protein)	1.76 ± 0.47	1.84 ± 0.28	0.746
SOD (U/mg protein)	25.60 ± 4.51	31.47 ± 3.64	0.032
CAT (U/mg protein)	119.80 ± 21.78	122.22 ± 11.36	0.462
GSH-Px (U/mg protein)	1336.93 ± 118.80	1268.70 ± 100.74	0.814
MDA (nmol/mg protein)	5.85 ± 3.30	5.15 ± 2.64	0.693
Fetal longissimus dorsi muscle			
T-AOC (U/mg protein)	0.40 ± 0.12	0.60 ± 0.13	0.022
SOD (U/mg protein)	23.72 ± 4.58	24.20 ± 4.53	0.860
CAT (U/mg protein)	139.48 ± 26.86	146.73 ± 20.76	0.613
GSH-Px (U/mg protein)	953.87 ± 183.85	1005.29 ± 159.79	0.616
MDA (nmol/mg protein)	6.00 ± 2.00	5.53 ± 1.43	0.651
